# Refractory gastrocutaneous fistula after gastrostomy removal successfully treated with submucosal dissection and endoscopic suture

**DOI:** 10.1055/a-2374-8564

**Published:** 2024-08-08

**Authors:** Alice Burgevin, Elena De Cristofaro, Claire Michoud, Emilien Daire, Lauriane Deker, Jean-Christophe Saurin, Mathieu Pioche

**Affiliations:** 1 Gastroenterology and Endoscopy Unit, Edouard Herriot Hospital, Hospices Civils de Lyon, Lyon, France; 2 9318 Gastroenterology Unit, Department of Systems Medicine, University of Rome Tor Vergata, Roma, Italy

Placement of a gastrostomy tube is a basic procedure involving the creation of a gastrocutaneous fistula through which a tube or button device is placed. The tube can be removed when access is no longer required for patients who recover from their disease. The tract starts to close within the first day and usually closes within 3 days. However, a fistula may persist in 25% of cases 1 . Surgical closure has been widely replaced by endoscopic techniques. Various techniques have been described in small patient series: argon plasma coagulation, silver nitrate, biopsy with forceps, endoscopic submucosal dissection or electrocautery can be used for de-epithelization of the fistulous tract. Fibrin glue, clips 2 , an over-the-scope clip 3 , or bands can be used for closure. Nevertheless, simple means are not always effective and sometimes the combination of mucosal dissection with submucosal closure was described to be effective after failure of standard treatments 4 .

Therefore, we progressively combine those two techniques from the first closure attempt to improve one-shot success 5 . We report the case of a 37-year-old patient with a persistent gastrocutaneous fistula 8 weeks after gastrostomy removal. We de-epithelialized the fistulous orifice using a submucosal dissection technique to promote healing and then closed the orifice with endoscopic suture (SutuArt; Olympus, Tokyo, Japan) ( Fig. 1 , Fig. 2 ) using the V-loc wire (Medtronic, Dublin, Ireland) ( Video 1 ). The patient was discharged on day 1 without any adverse event and the gastrocutaneous fistula was completely healed at the 4-month clinical re-evaluation.

**Fig. 1:**
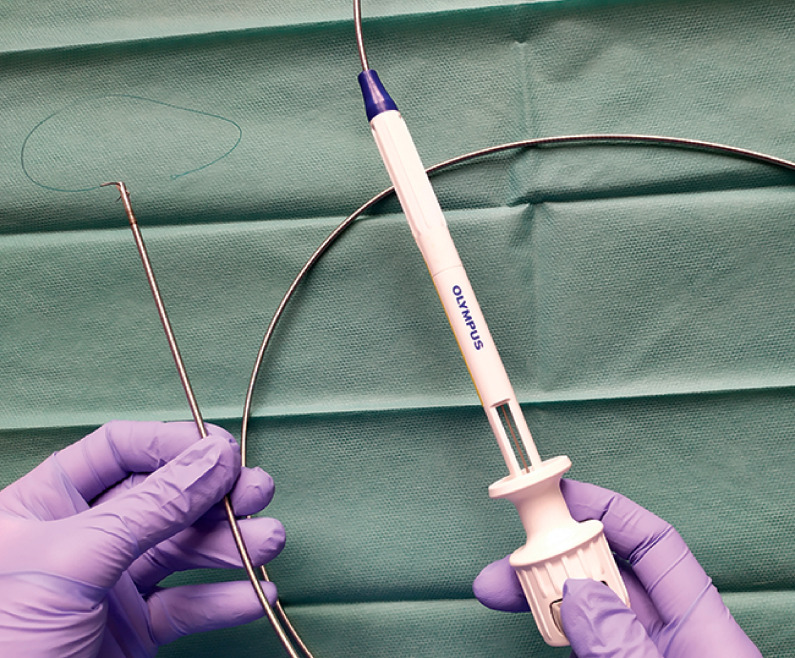
SutuArt needle (Olympus, Tokyo, Japan).

**Fig. 2:**
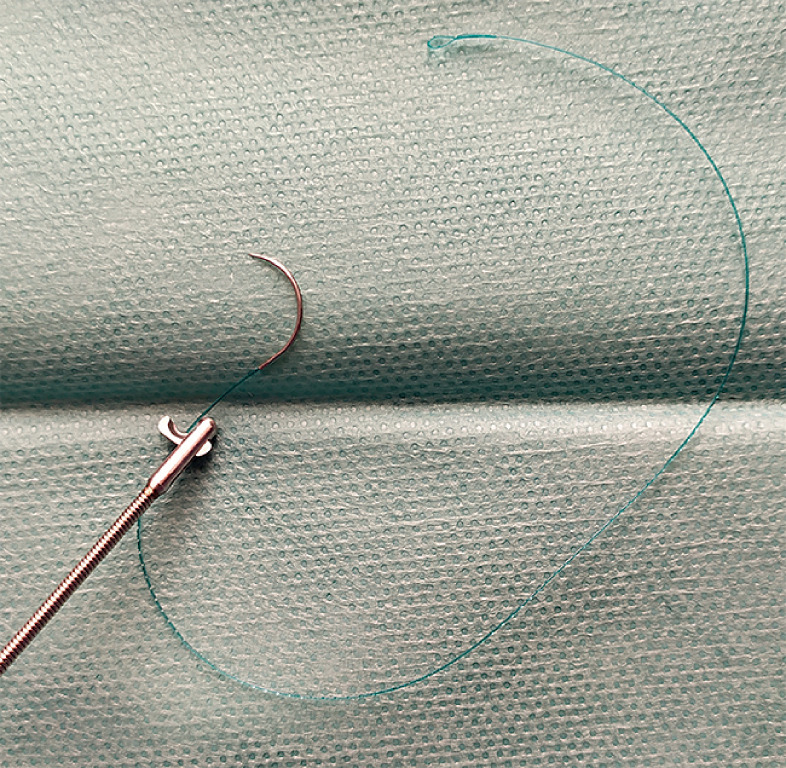
Endoscopic scissors (Olympus, Tokyo, Japan).

Procedure of endoscopic submucosal dissection combined with endoscopic suture of a gastrocutaneous fistula after percutaneous endoscopic gastrostomy tube removal.Video 1

We can assume that this technique may be a therapeutic option for refractory gastro-cutaneous fistulas.

Endoscopy_UCTN_Code_TTT_1AO_2AO

## Notes

Endoscopy E-Videos https://eref.thieme.de/e-videos
*E-Videos* is an open access online section of the journal *Endoscopy* , reporting on interesting cases and new techniques in gastroenterological endoscopy. All papers include a high-quality video and are published with a Creative Commons CC-BY license. Endoscopy E-Videos qualify for HINARI discounts and waivers and eligibility is automatically checked during the submission process. We grant 100% waivers to articles whose corresponding authors are based in Group A countries and 50% waivers to those who are based in Group B countries as classified by Research4Life (see: https://www.research4life.org/access/eligibility/ ). This section has its own submission website at https://mc.manuscriptcentral.com/e-videos .
